# Highly Pathogenic H5N1 Influenza A Virus (IAV) in Blue-Winged Teal in the Mississippi Flyway Is Following the Historic Seasonal Pattern of Low-Pathogenicity IAV in Ducks

**DOI:** 10.3390/pathogens13111017

**Published:** 2024-11-19

**Authors:** David E. Stallknecht, Deborah L. Carter, Lyndon Sullivan-Brügger, Paul Link, Emily Ferraro, Ciara McCarty, Bruce Davis, Lynda Knutsen, James Graham, Rebecca L. Poulson

**Affiliations:** 1 Southeastern Cooperative Wildlife Disease Study, Department of Population Health, College of Veterinary Medicine, University of Georgia, Athens, GA 30602, USA; 2 Louisiana Department of Wildlife and Fisheries, 5476 Grand Chenier Hwy, Grand Chenier, LA 70643, USA; 3 School of Renewable Resources, Louisiana State University Agricultural Center, Baton Rouge, LA 70803, USA; 4 Wetland Wildlife Populations and Research Group, Minnesota Department of Natural Resources, Bemidji, MN 56601, USA; 5 Agassiz National Wildlife Refuge, U.S. Fish and Wildlife Service, Middle River, MN 56737, USA

**Keywords:** antibody, dabbling ducks, HP H5N1, population immunity, seasonality, seroprevalence

## Abstract

Highly pathogenic H5N1 (HP H5N1) influenza A virus (IAV) has been detected annually in North American ducks since its introduction during 2021, but it is unknown if this virus will follow the same seasonal and geographic patterns that have been observed with low-pathogenicity (LP) IAV in this reservoir. We monitored blue-winged teal in the Mississippi flyway prior to the detection of HP H5N1 and during two post-introduction migration cycles from spring 2022 to spring 2024, testing birds for infection and antibodies to IAV nucleoprotein (NP), hemagglutinin subtype H5, and neuraminidase subtype N1. Antigens representing clade 2.3.4.4b HP H5 and LP North American H5 were used for hemagglutination inhibition (HI) and virus neutralization (VN) tests for H5 antibodies. Virologic results were consistent with historic seasonal and geographic patterns reported for LP IAV with peak infections occurring in pre-migration staging areas in Minnesota during fall 2022. However, the high prevalence of the H5 subtype was exceptional compared to historic prevalence estimates at this same site and for the Mississippi flyway. HP H5N1 was detected on wintering areas in Louisiana and Texas during the fall of that same year and this was followed by an increase in estimated antibody prevalence to NP, H5, and N1 with no HP H5N1 detections during the wintering or spring migration periods of 2022/2023. HP H5N1 was not detected in Minnesota during fall 2023 but was detected from a single bird in Louisiana. However, a similar increase in antibody prevalence was observed during the winter and spring period of 2023 and 2024. Over the two migration cycles, there was a temporal shift in observed prevalence and relative titers against the H5 antigens with a higher proportion of ducks testing positive to the 2.3.4.4b H5 antigen and higher relative titer to that antigen compared to the representative LP North American H5 antigen. The seasonal and geographic patterns observed appear to be driven by population immunity during the migration cycle. Results support an initial high infection rate of HP H5N1 in blue-winged teal in the Mississippi flyway followed by a high prevalence of antibodies to NP, H5, and N1. Although prevalence was much reduced in the second migration cycle following introduction, it is not known if this pattern will persist in the longer term or affect historic patterns of subtype diversity in this reservoir.

## 1. Introduction

In December 2021, clade 2.3.4.4b A/goose/Guangdong/1/1996 (Gs/GD) H5N1 highly pathogenic (HP) influenza A virus (IAV) (HP H5N1) was detected in eastern Canada [1]. This and additional introductions of HP H5N1 from both Europe and Asia during 2022 resulted in a rapid spread to numerous and diverse wild bird species extending from North America to Antarctica [2,3,4]. Since 2022, HP H5N1 has been detected in North American wild birds every year with numerous infections occurring in ducks [5].

Although the natural history of endemic low-pathogenicity (LP) IAV in North American waterfowl is well described, it is unknown if this knowledge can be applied to seasonal or geographic patterns that may occur with HP H5N1. With endemic LP IAV, the prevalence of IAV infection varies greatly by waterfowl species and season, with peak infection prevalence in most dabbling duck species occurring in juvenile (hatch year) birds during pre-migration staging in northern North America during late summer and early fall [6,7,8]. The prevalence of IAV infection in duck populations decreases during fall migration and winter, and this has been attributed to increased population immunity to IAV [9]. An additional but relatively small increase in the prevalence of IAV infection in ducks can also occur during spring migration [8,10]. Infections in North American waterfowl are also characterized by the presence of numerous IAV subtypes, primarily representing hemagglutinin (HA) H1-12 and neuraminidase (NA) N1-N9 in ducks [7]. Many combinations of these HA/NA subtypes occur in duck populations as a result of reassortment, but subtype diversity is dominated by certain combinations, such as H3N8 and H4N6, which are consistently over-represented each year, especially during late summer and early fall [7,11]. In contrast, some subtypes, such as H7 and H10, are commonly detected during spring migration [10,12]. It is possible that subtype diversity and seasonal dominance of specific subtypes also are partially driven by population immunity, but this has not been adequately evaluated.

Host-related factors, which allow or prevent a new HP IAV to successfully enter and persist in wild bird populations already dominated by high rates of infection with naturally occurring LP IAV, are undefined. The immunologic status of waterfowl populations prior to an introduction is one of these potential factors, but such data are not currently available for North American waterfowl populations immediately prior to the 2021 introduction of HP H5N1. This information could provide insight into conditions that facilitate the successful invasion of an HP H5N1 to a new ecosystem or avian population and the longer-term trajectory of a successful introduction. Introductions are not always successful, as evident from the failure of 2014 HP H5Nx to persist in North American waterfowl populations [13].

The need to include population immunity as one of the host factors that may affect the outcome of an HP IAV introduced into a wild bird population is supported by both field and experimental studies involving host response to controlled sequential IAV infections. Field studies have demonstrated annual shifts in subtype diversity possibly related to cyclic patterns of population immunity [11,14,15]. Subtype-specific protection consistent with homosubtypic and heterosubtypic immunity also has been demonstrated in long-term field studies of mallards (*Anas platyrhynchos*) in Sweden [16]. HP H5N1 mortality in mute swans (*Cygnus olor*) was also reduced in adult birds attributable to pre-existing immunity [17]. Results from experimental studies with both LP and HP IAV sequential challenges indicate that previous infections with LP IAV in ducks can result in reduced infection, mortality, and viral shedding during subsequent infections with both LP and HP IAVs and can also increase the infective dose required for challenge infection [16,18,19,20,21,22,23]. These effects, which could potentially affect transmission, are more pronounced with genetically similar or matched HAs and repeated LP IAV infections [22,24]. With regard to potential population immunity barriers that could affect the outcome of an introduced Gs/GD HP H5 lineage IAV into North America, it has been clearly demonstrated that prior infection with LP H5 IAV and less virulent strains of HP Gs/GD H5N8 can also result in reduced shedding and clinical effects [1,25]. Immunity directed at NA also could be an important component of population immunity. Although NA is an important antigen affecting the IAV susceptibility of mice and humans, NA immunity has not been adequately evaluated in either field or experimental studies directed at waterfowl [26].

In this study, we investigate the seasonal and geographic prevalence of HP H5N1 infections in ducks in the Mississippi flyway preceding and following its introduction. We also employ multiple serologic tests to detect antibodies to IAV nucleoprotein (NP), and we use H5 and N1 to explore the potential relationship between infection prevalence and population immunity over seasons and years. Specific objectives are to determine the following: (1) if the seasonal, geographic, and age-related patterns of HP H5N1 infection in ducks follow the historic patterns reported for LP IAV in North America; (2) if antibodies to H5 and N1 are detectable in ducks over the annual cycle and potentially correlate with HP H5N1 prevalence; (3) if detected H5 and N1 antibodies from ducks can be related to previous infections with HP H5N1 or endemic LP IAV with these same H5 and N1 subtypes; and (4) if seasonal infection and serologic patterns observed in blue-winged teal (*Spatula discors*) are also observed in other dabbling duck species sampled at the same time and locations.

## 2. Materials and Methods

### 2.1. Sample Collection

Ducks were captured and sampled at multiple locations in Louisiana, Texas, and Minnesota from April 2022 to April 2024 (Table S1). Blue-winged teal were the primary species tested but mallard and green-winged teal (*Anas carolinensis*) were also sampled for species comparisons. Blue-winged teal and mallard were captured in Minnesota during September 2022 and 2023 as part of banding operations carried out by personnel from the Minnesota Department of Natural Resources at the Thief Lake Wildlife Management Area (WMA; Marshall County; 48.486903, −95.950603) and Roseau River WMA (Roseau County; 48.977714, −96.008911) or the United States Fish and Wildlife Services (USFWS) at the Agassiz National Wildlife Refuge (Marshall County; 48.300806, −95.980467) in northwestern Minnesota, USA. Ducks captured on wintering grounds and during spring migration were collected as part of spring banding efforts in collaboration with personnel from the Louisiana Department of Wildlife and Fisheries. When possible, birds were aged based on plumage characteristics and classified as juvenile or adult. Juvenile birds represented birds hatched in early summer and sampled through the following spring. Adults represented the breeding population of birds present in the population through the following spring. All samples were collected with appropriate states (LA WDP-22-018, WDP-24-017; MN 35074, 32793; and TX SPR-1022-137) and federal (USFWS MB53692D) permits.

Hunter harvested birds were also tested for HP H5N1 infection in January and September 2022 in Louisiana and Texas and during September 2023 in Louisiana. In addition to blue-winged teal, the testing of hunter-killed birds in Louisiana in January 2022 also included American wigeon (*Mareca americana*), black-belied whistling duck (*Dendrocygna autumnalis*), bufflehead (*Bucephala albeola*), fulvous whistling duck (*D. bicolor*), gadwall (*M. strepera*), lesser scaup (*Aythya affinis*), mottled duck (*Anas fulvigula*), northern pintail (*Anas acuta*), northern shoveler (*S. clypeata*), redhead (*Aythya americana*), and ring-necked duck (*Aythya collaris*). In January 2023, northern pintails were also sampled during hunter harvest in Louisiana. All birds were aged based on plumage characteristics.

Paired cloacal/oropharyngeal (CL/OP) swabs from each duck were combined in a cryovial with 2 mL of Brain Heart Infusion broth (Becton Dickinson and Co., Sparks, MD, USA) supplemented with penicillin G (1000 units/mL), streptomycin (1 mg/mL), kanamycin (0.5 mg/mL), gentamicin (0.25 mg/mL), and amphotericin B (0.025 mg/mL) (Sigma Chemical Company, St. Louis, MO, USA). All samples were stored in the field at 4 °C, shipped to the lab within seven days of sampling, and stored at −70 °C until testing. Blood samples (≤1 mL) from blue-winged teal and mallard were collected via jugular or brachial venipuncture. Sera were stored at 4 °C in the field, heat-inactivated at 56 °C for 30 min upon receipt in the laboratory, and frozen at −20 °C until testing.

### 2.2. HP H5N1 Detection

All CL/OP swabs were either tested via a real-time reverse transcription polymerase chain reaction (rrt-PCR) targeting 2.3.4.4b H5 or inoculated into embryonated chicken eggs (ECEs) for virus isolation (VI), as previously described [27]. Swabs tested directly by rrt-PCR were first extracted using a MagMax-96 AI/ND Viral RNA Isolation Kit (Applied Biosystems, Foster City, CA, USA) on the Thermo Electron KingFisher magnetic particle processer (Thermo Electron Corporation, Waltham, MA, USA) [28]. Resultant viral RNAs were screened in rrt-PCR against primers specific for 2.3.4.4b H5; all non-negatives with cycle thresholds (Ct) <40 in this assay were sent to the United States Department of Agriculture (USDA) National Veterinary Services Laboratory (NVSL) to confirm the subtype and pathogenicity. When VI was attempted, amnio-allantoic fluids were collected from ECE after 96 h of incubation at 36 °C and tested via the hemagglutination assay [29]. Viral RNA was extracted (QIAamp Viral RNA Mini Kit, Qiagen Inc., Valencia, CA, USA) from all hemagglutination assay-positive samples, as per the manufacturer’s instructions, and tested against primers specific for the 2.3.4.4b clade of H5 IAV in rRT-PCR; samples that yielded a Ct value < 40 were submitted to the USDA NVSL for confirmatory testing.

### 2.3. Serology

Sera were tested for antibodies to IAV NP using a commercial bELISA (IDEXX AI MultiS-Screen AB Test, IDEXX Laboratories, Westbrook, ME, USA). Samples testing positive with a negative absorbance ratio (S/N) of <0.7 were further tested by hemagglutination inhibition (HI), virus neutralization (VN), and an enzyme-linked lectin assay (ELLA) for antibodies to H5 and N1, as previously described [30,31]. Two attenuated viruses produced by reverse genetics (rg) were used as antigens for HI and VN, specifically rg BWT containing the HA and NA from the LP A/blue-winged teal/AI12-4150/Texas/2012 (H5N2) (hereafter LP rg BWT) and IDCDC-RG71A (H5N8) (hereafter HP rg AST) containing a modified HA and NA from the HP 2.3.4.4b A/Astrakhan/3212/2020 (H5N8); the remaining gene segments from both viruses were obtained from A/Puerto Rico/8/34. For ELLA, A/ruddy turnstone/New Jersey/AI13-2948/2013 (H10N1) was used as the antigen. Samples were considered positive for antibodies to H5 if they tested positive for either antigen at a titer of 32 or 20 for HI and VN, respectively. For ELLA, a titer of 80 was considered positive.

## 3. Results

### 3.1. HP H5N1 Detection

**Figure 1:**
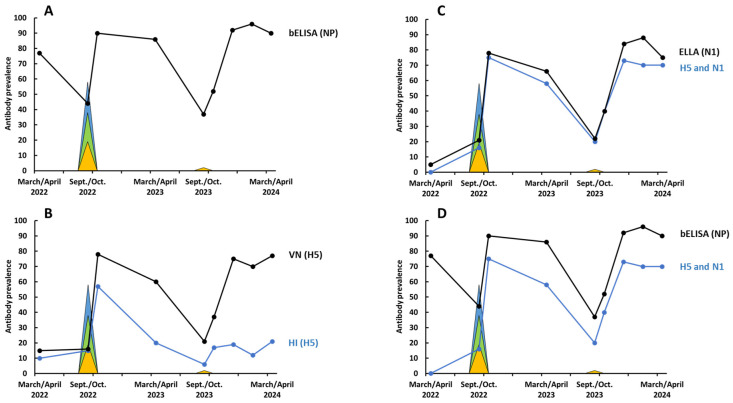
Estimated prevalence of antibodies in blue-winged teal sampled from March 2022 to April 2024: (**A**) antibodies to nucleoprotein (NP), as detected with bELISA; (**B**) antibodies to H5, as detected by hemagglutination inhibition (HI) and virus neutralization (VN); (**C**) antibodies to N1, as detected by enzyme-linked lectin assay (ELLA) and the percentage of teal testing positive for antibodies to both H5 and N1; (**D**) the percentage of teal testing positive for antibodies to both H5 and N1 compared to those testing antibody-positive to all subtypes based on NP. Filled color areas represent the prevalence of HP H5N1 infection detected in blue-winged teal sampled in Minnesota (blue) and Louisiana/Texas (gold) and mallards sampled in Minnesota (green).

From January 2022 to April, 2024, 3791 birds were tested for HP H5N1 infection (Table S1; Figure 1A). Although HP H5N1 was detected as far south as Tennessee in the Mississippi flyway during January 2022 [32,33], we did not detect HP H5N1 in 1103 ducks sampled in Louisiana from January to April 2022. During staging and early fall migration in Minnesota during September 2022, HP H5N1 was detected by virus isolation or rrt-PCR in 134 of 233 (58%) and 28 of 73 (38%) sampled blue-winged teal and mallards, respectively. Early migrating blue-winged teal on wintering grounds in Texas and Louisiana were also infected with HP H5N1 during September 2022, where 70 of 309 (23%) and 57 of 352 (16%) tested positive, respectively. We did not detect HP H5N1 in 155 ducks sampled on wintering grounds in Louisiana from October 2022 to January 2023 or in 284 spring-migrating blue-winged teal sampled in Louisiana during March and April 2023. During staging and fall migration sampling (September 2023), HP H5N1 was not detected in 79 blue-winged teal and 129 mallards sampled in Minnesota, but 1 of 302 (<1%) blue-winged teal tested positive for HP H5N1 during sampling on winter in grounds in Louisiana. None of the 339 samples collected from wintering ducks in Louisiana from October 2023 to February 2024 were infected. Likewise, HP H5N1 was not detected in 433 spring-migrating blue-winged teal sampled in Louisiana during March and April 2024.

### 3.2. Antibodies in Blue-Winged Teal

From April 2022 to April 2024, 1348 blue-winged teal were tested for antibodies to IAV, H5, and N1 (Table S1). Antibodies to IAV in blue-winged teal, as determined by bELISA, were detected at all locations and sample periods, but varied greatly between seasons and migration statuses (Figure 1A). During spring migration from March to April 2022, and prior to the detection of HP H5N1, 143 of 185 (77%) blue-winged teal tested positive for antibodies to IAV. In September 2022, with the recruitment of juvenile blue-winged teal, IAV antibody prevalence decreased to 44% but increased rapidly following the detection of HP H5N1. By spring 2023, an estimated 85% of the population had antibodies to IAV. This same seasonal trend in IAV antibody prevalence was observed during the fall 2023/spring 2024 seasonal migration cycle (Figure 1A).

**Figure 2:**
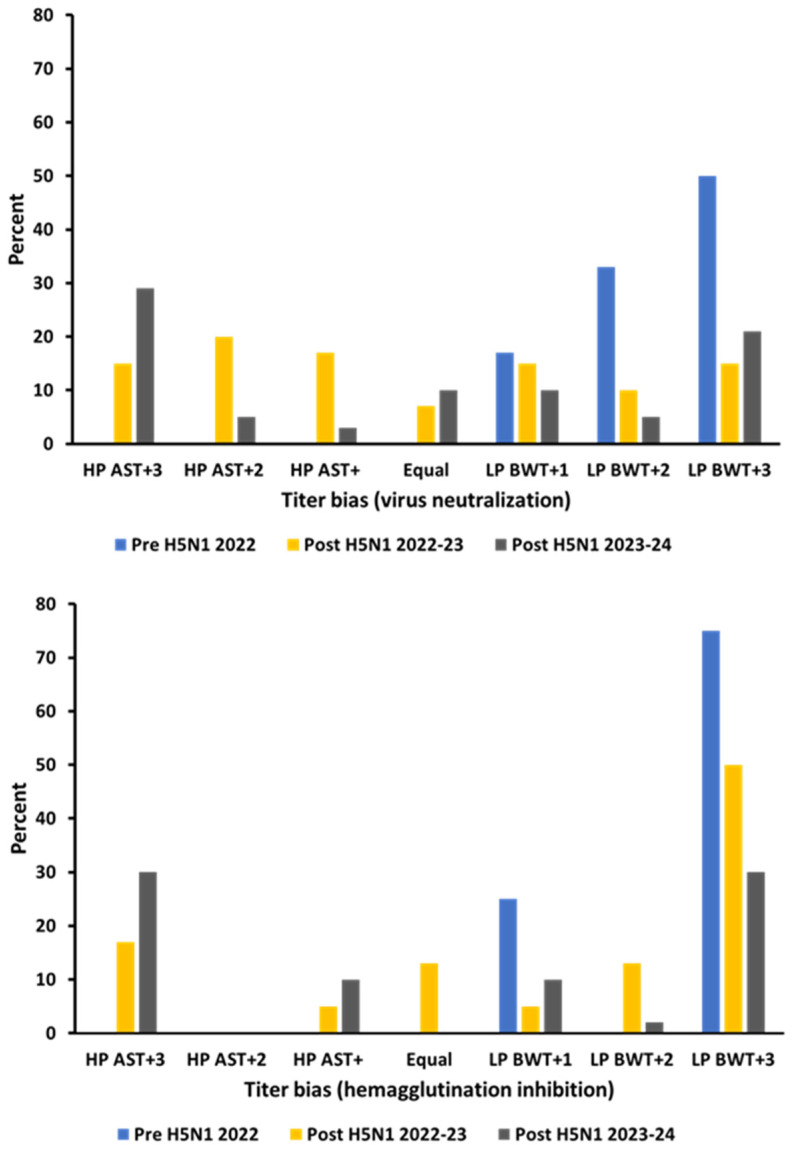
Difference in antibody titers to two reverse genetic viruses with HA and NA from HP 2.3.4.4b A/Astrakhan/3212/2020 (H5N8) and (HP AST) LP A/blue-winged teal/AI12-4150/Texas/2012(H5N2) (LP BWT), as determined by virus neutralization and hemagglutination inhibition. Numbers (+1 to +3) represent titer differences (number of two-fold dilutions biased towards HP AST or LP BWT).

Antibodies to H5 were detected by both HI and VN (Figure 1B). Although H5 antibody prevalence estimates for blue-winged teal were consistently lower for HI than VN, seasonal trends were similar (Figure 1B). Of the total 94 blue-winged teal samples testing HI positive for H5 antibodies, 91 (97%) also tested positive using VN. During pre-migration staging from March to April 2022, and prior to the detection of HP H5N1, the estimated prevalence rates of antibodies to H5 in blue-winged teal, as determined by HI and VN, were 10% and 15%, respectively. All of these HI and VN seropositive samples tested positive to the North American LP BWT antigen only (Table 1). With H5, estimated antibody prevalence followed the same seasonal pattern observed with IAV (NP) antibody prevalence and was lowest during fall premigration staging in Minnesota during both 2022 (HI = 15% and VN = 16%) and 2023 (HI = 6% and VN = 21%) (Figure 1B). Unlike results from the spring 2022 samples, which presumably were collected prior to HP H5N1 introduction into Louisiana, positive results to both the LP rg BWT and the HP rg AST antigens were consistently detected by HI and VN in all subsequent sampling periods (Table 1). While most H5 antibody-positive samples tested positive to both antigens, the proportions of samples testing H5 antibody-positive only for HP rg AST increased over time for both HI and VN (Table 1). Likewise, the percentage of samples testing positive only for LP rg BWT antigens decreased over this same time period (Table 1). A similar temporal change was apparent when comparing HI and VN antibody titers for these two antigens where an antigenic shift towards the HP rg AST antigen was observed over time (Figure 2).

Antibodies to N1 were detected in all sample periods and the estimated prevalence followed the same seasonal patterns of the estimated prevalence of IAV (NP) and H5 with seasonal lows occurring in the premigration Minnesota samples (Figure 1C). As observed with antibody prevalence estimates for H5, the prevalence of N1 in the pre-detection HP H5N1 sample period in Louisiana (March/April 2022) was the lowest observed (5%) over the entire study. In March/April 2023 and 2024, the estimated prevalence of antibodies to N1 increased to 66% and 75%, respectively. Most of the N1 seropositive IAV (NP)-positive ducks also tested seropositive for antibodies to H5 with seasonal prevalence peaks observed in the winter and spring samples (Figure 1C). Of the 351 IAV NP-positive samples detected from September/October 2022 to March April/2024, 241 (69%) tested seropositive for both H5 and N1. In contrast, none of the 30 IAV (NP) antibody-positive blue-winged teal tested during the initial March/April 2022 sample period tested positive for antibodies to both H5 and N1.

### 3.3. Species/Migration Status/Age Comparisons

Mallards (*n* = 166) sampled at the same times and locations as blue-winged teal in Minnesota during September 2022 and 2023 were more likely than blue-winged teal to test positive for antibodies to both H5 and N1 (Table 2). For both mallards and blue-winged teal, more adult birds tested antibody-positive for both H5 and N1 than juvenile birds. One exception was observed (blue-winged teal, Louisiana, December 2023) where prevalence estimates were slightly higher in juvenile birds. For blue-winged teal, a higher proportion of birds tested positive for antibodies to both H5 and N1 during the wintering and spring period than observed on premigration staging areas in Minnesota, with the greatest difference observed in the juvenile cohort. Green-winged teal (*n* = 27) were sampled with blue-winged teal during January and February 2024 in Louisiana (Table S1). Approximately 78% and 56% of adult blue-winged teal and green- winged teal, respectively, tested seropositive for both H5 and N1.

## 4. Discussion

HP H5N1 entered the Mississippi flyway during January 2022 but was not detected in our initial testing of 1103 ducks sampled in Louisiana from January to April 2022 and seemingly did not reach waterfowl wintering areas that far south during spring 2022. Serologic results from 95 blue-winged teal sampled during this presumptive HP H5N1 pre-detection period in March/April 2022 are consistent with negative virologic results showing a high (77%) estimated prevalence of antibodies to IAV (NP) but low prevalence estimates for H5 (HI = 10% and VN = 15%) and N1 (5%). The high prevalence of antibodies to IAV is normal for spring-migrating blue-winged teal in Louisiana, and, during March/April 2015 and 2016, prevalence was estimated at 61% and 66%, respectively [31]. The low prevalence of H5 and N1 antibodies and the observed H5 titer bias towards the representative North American LP H5 antigen (rg BWT) (Figure 2) are all consistent with an absence or very low prevalence of HP H5N1 in the lower wintering grounds of the Mississippi flyway during Spring 2022.

The prevalence of LP H5 IAV infection in ducks can vary significantly between years, as determined by studies in Alberta and Minnesota [14,34]. This annual variation has also been documented with H5 antibody prevalence estimates from mallards sampled in Minnesota during September which annually varied from 5% to 38% in juvenile birds and from 61% to 100% in adult mallards sampled from 2014 to 2018 [31]. Based on reported results from previous experimental studies, pre-existing immunity to IAV, but not H5, could potentially reduce shedding and transmission but should not prevent infection with HP H5N1 [16,18,19]. If our results from blue-winged teal are representative of other duck species and populations in the Mississippi flyway during the 2021/2022 wintering and spring migration seasons, limited detectable immunity to H5 and possibly N1 during the 2021/2022 migration cycle could have facilitated the successful introduction of HP H5N1 into North America during that winter.

Historically, the prevalence rates of IAV and LP H5 IAV infection are low in the Mississippi flyway during winter and spring. Based on long-term data collected from 1976 to 2015, IAV infections, including all subtypes, were detected in only 14% of 9507 and 7% of 5841 waterfowl sampled during winter and spring, respectively [7]. These IAV isolates included only 14 LP H5 IAVs during winter and 2 LP H5 IAVs during spring. In winter and spring of 2022, immediately following HP H5N1 introduction, this pattern was not observed, with HP H5N1 routinely detected in North American ducks, including ducks from the Mississippi flyway [5,33]. Following this initial outbreak period, HP H5N1 prevalence estimates observed in blue-winged teal during migratory seasons (fall 2022–spring 2023 and fall 2023–spring 2024) followed more recognized seasonal and geographic patterns consistent with LP IAV in ducks. In these following years, most of our HP H5N1 detections were associated with September pre-migration staging/early fall migration areas in Minnesota. During the first fall migration (2022) following HP H5N1 introduction, HP H5N1 prevalence rates were estimated as 58% and 38% in blue-winged teal and mallards, respectively, at this site. During this same migration season, the virus was also detected in blue-winged teal on wintering areas in Louisiana and Texas with an estimated prevalence of 16% and 23%, respectively. The high prevalence of IAV infection on northern breeding and staging areas such as Minnesota is normal, and it is not uncommon to detect IAV at a lower prevalence on wintering grounds, especially in early-migrating blue-winged teal sampled in September [10,27].

Although HP H5N1 seemingly followed previously documented LP IAV seasonal and geographic patterns in the two migratory fall/spring seasons following introduction, our virus isolation and serologic data both suggest that HP H5N1 infection was much more prevalent than historic data for LP H5 would predict. Of 408 IAVs detected at this study site in Minnesota during 2007 and 2008, only 15 LP H5 IAVs were isolated [34]. Similar results are reported from ducks in Alberta sampled in August and September from 1976 to 1990 [35]. Likewise, of 650 LP IAVs isolated from blue-winged teal in Louisiana from 2007 to 2017, only 4 LP H5 IAVs were detected [10]. This high prevalence of infection observed with HP H5N1 is not currently understood and probably relates to many interacting factors, including very efficient replication in ducks, high transmissibility, and possibly a low level of H5- and N1-specific population immunity prior to introduction.

Although it has long been accepted that the seasonal pattern of LP IAV infection is associated with population immunity, supporting serologic evidence is limited. The seasonal trends for antibodies to NP (Figure 1A), H5 (Figure 1B), N1 (Figure 1C), and H5 and N1 (Figure 1D) all are consistent with virologic results, with HP H5N1 detection identified during early-fall sampling periods with the lowest observed prevalence of antibodies to IAV, H5, and N1. The importance of annual recruitment to possible long-term HP H5N1 seasonality is also apparent from our data. Differences in antibody prevalence estimates observed during fall migration and more similar estimates during winter are consistent with seasonal differences in LP IAV infection prevalence observed in juvenile and adult ducks [36]. In both the 2022 and 2023 fall migrations, the detection of HP H5N1 was followed by an overall increase in antibody prevalence (NP, H5, and N1), with no additional detections of this virus during winter or spring sampling. In 2023, only one HP H5N1 virus was isolated. However, this low prevalence of infection was not consistent with the increase in antibodies to H5 and N1 observed during later sampling. Annual variations in the timing of peak IAV infection in ducks at the Minnesota study site do occur [34] and it is possible that we sampled ducks in Minnesota before HP H5N1 entered this site.

Although data from this study are limited, results from mallards and green-winged teal sampled at the same time and locations as blue-winged teal demonstrated consistent results for virus isolation and serology. The serologic testing of additional dabbling duck species and waterfowl taxa is warranted, as differences in migratory timing, behavior, population age structure, and possibly susceptibility may affect spatial and temporal patterns of infection.

The serology testing approach utilized in this study provided a novel perspective on the seasonality and extent of HP H5N1 infection in the Mississippi flyway, but limitations relative to data interpretation exist. For HP H5N1, we do not know the duration of the detectable antibody response to NP, H5, or N1, and there is limited information to provide direction related to correlates of protection associated with these antibodies. In an experimental sequential infection, antibodies to NP were detectable in only 33% of mallards previously infected with HP H5N8; however, even in the absence of a detectable NP response, ducks were protected upon challenge [1]. In this same study, more tufted ducks (*Aythya fuligula*) were protected from clinical disease. It is also difficult to translate experimental results to the field. In our study (Figure 1A), and in a previous study with wild sentinel mallards in Alaska [37], a loss of or reduction in NP antibody prevalence during winter and spring following peak HP H5N1 during fall was not detectable or minimal. This difference probably relates to recurring challenges with HP H5N1 and other LP IAV subtypes that occur in the field. We did observe declines in antibody prevalence in winter and spring during the 2022/2023 migration season for H5, as determined by both HI and VN (Figure 1B) and N1 (Figure 1C). Such a decline was not readily apparent during the 2023/2024 migratory season.

The interpretation of test and antigen-specific titers also needs to be further investigated. Our antibody prevalence and titer comparisons for representative LP and HP antigens both provided results that were consistent with an increase in HP H5N1 infections over time but also demonstrate the difficulty of interpreting such data from birds naturally infected in the field. Although serologic results suggest that a high proportion of ducks were infected with HP H5N1 during both migratory seasons, a higher titer (two or more dilutions) for HP rg AST compared to LP rg BWT was observed in relatively few birds, as determined by both HI and VN (Figure 2). How imprinting or repeated infections with LP IAV potentially affect such antigenic comparisons is not well understood. With regard to test results, we consistently observed a lower antibody prevalence and titer with HI compared to VN. Part of this may have related to using an HI cutoff value of 32, which may have resulted in good agreement (95%) between positive HI and VN results but an underestimate of HI antibody prevalence. The lack of a robust HI antibody response in mallards experimentally and naturally infected with IAV has been previously reported [38,39].

Even with these limitations, serologic testing represents an additional tool that, when possible, should be considered to support field studies. Our results provide evidence that population immunity following an HP H5N1 outbreak can be detected, and, when prevalence to H5 and N1 antibodies is high, infection rates are low. With additional work to improve serologic data interpretation and testing approaches, serologic testing may provide a valuable tool to better understand seasonal and geographic risks of HP H5N1 introduction and spillover to other species, identify wildlife species or populations at risk, and better understand and perhaps predict the impacts and outcomes of infections.

## Figures and Tables

**Table 1:** Proportion of samples from blue-winged teal testing positive (+) on hemagglutination inhibition and virus neutralization tests for H5 antibodies to two reverse genetic viruses representing clade 2.3.4.4b HP H5 (rg AST) and North American LP H5 (rg BWT).

Test	H5 Antibody-Positive Results Based on Antigens	Pre-Detection HP H5N1 (2022)	First Migration Season (Fall 2022–Spring 2023)	Second Migration Season (Fall 2023–Spring 2024)
Hemagglutination inhibition	+HP rg AST only	0 (0%)	9 (17%)	9 (25%)
	+HP rg AST and + LP rg BWT	0 (0%)	15 (28%)	9 (25%)
	+LP rg BWT only	4 (100%)	30 (55%)	18 (50%)
	Total H5-positive	4	54	36
Virus neutralization	+HP rg AST only	0 (0%)	15 (17%)	35 (23%)
	+HP rg AST and +LP rg BWT	0 (0%)	46 (52%)	72 (48%)
	+LP rg BWT only	6 (100%)	27 (31%)	44 (29%)
	Total H5-positive	6	88	151

**Table 2:** Estimated prevalence for antibodies to both H5 and N1 by migration status, species, and age.

Migration Status	Pre-Migration Staging/Fall Migration				Wintering	
Species	Blue-Winged Teal		Mallard		Blue-Winged Teal	
Month/year	Sept/2022	Sept/2023	Sept/2022	Sept/2023	Dec/2023	Jan–Feb/2024
Location	Minnesota	Minnesota	Minnesota	Minnesota	Louisiana	Louisiana
Juvenile ^A^	10% ^B^	4%	46%	11%	78%	56%
Adult	44%	74%	100%	78%	72%	78%

^A^ Juvenile: sampled within one year of hatch; adult: sampled during second year or more after hatch. ^B^ Estimated percentage of sampled birds testing positive for antibodies to both H5 and N1 = proportion of total samples testing positive for antibodies IAV (bELISA) × proportion of IAV-seropositive birds testing positive for antibodies to H5 and N1.

## Data Availability

All data in support of this manuscript are included in the text or in the Supplementary Materials.

## Supplementary Materials

The following supporting information can be downloaded at: https://www.mdpi.com/article/10.3390/pathogens13111017/s1. Table S1: 2.3.4.4b H5 virus detection data based on virus isolation and/or rrt-PCR, and antibody detection based on bELISA; hemagglutination inhibition (HI) and virus neutralization (VN) which were used to test for antibodies to H5; and enzyme-linked lectin assay (ELLA) which was used to test for antibodies to N1 and for ducks sampled in the Mississippi flyway of the United States from January 2022 to April 2024. Data are compiled by data, state, species, and age.
